# Multiparametric MRI Radiomics for the Early Prediction of Response to Chemoradiotherapy in Patients With Postoperative Residual Gliomas: An Initial Study

**DOI:** 10.3389/fonc.2021.779202

**Published:** 2021-11-18

**Authors:** Zhaotao Zhang, Keng He, Zhenhua Wang, Youming Zhang, Di Wu, Lei Zeng, Junjie Zeng, Yinquan Ye, Taifu Gu, Xinlan Xiao

**Affiliations:** ^1^ Department of Radiology, The Second Affiliated Hospital of Nanchang University, Nanchang, China; ^2^ Department of Radiology, Hsiang-ya Hospital, Changsha, China; ^3^ Department of Radiology, The First Affiliated Hospital of Gannan Medical College, Ganzhou, China; ^4^ Department of Oncology, The Second Affiliated Hospital of Nanchang University, Nanchang, China; ^5^ Department of Radiology, The Fifth Affiliated Hospital of Jinan University, Heyuan, China

**Keywords:** radiomics, magnetic resonance imaging, residual gliomas, chemoradiotherapy, early prediction

## Abstract

**Purpose:**

To evaluate whether multiparametric magnetic resonance imaging (MRI)-based logistic regression models can facilitate the early prediction of chemoradiotherapy response in patients with residual brain gliomas after surgery.

**Patients and Methods:**

A total of 84 patients with residual gliomas after surgery from January 2015 to September 2020 who were treated with chemoradiotherapy were retrospectively enrolled and classified as treatment-sensitive or treatment-insensitive. These patients were divided into a training group (from institution 1, 57 patients) and a validation group (from institutions 2 and 3, 27 patients). All preoperative and postoperative MR images were obtained, including T1-weighted (T1-w), T2-weighted (T2-w), and contrast-enhanced T1-weighted (CET1-w) images. A total of 851 radiomics features were extracted from every imaging series. Feature selection was performed with univariate analysis or in combination with multivariate analysis. Then, four multivariable logistic regression models derived from T1-w, T2-w, CET1-w and Joint series (T1+T2+CET1-w) were constructed to predict the response of postoperative residual gliomas to chemoradiotherapy (sensitive or insensitive). These models were validated in the validation group. Calibration curves, receiver operating characteristic (ROC) curves, and decision curve analysis (DCA) were applied to compare the predictive performances of these models.

**Results:**

Four models were created and showed the following areas under the ROC curves (AUCs) in the training and validation groups: Model-Joint series (AUC, 0.923 and 0.852), Model-T1 (AUC, 0.835 and 0.809), Model-T2 (AUC, 0.784 and 0.605), and Model-CET1 (AUC, 0.805 and 0.537). These results indicated that the Model-Joint series had the best performance in the validation group, followed by Model-T1, Model-T2 and finally Model-CET1. The calibration curves indicated good agreement between the Model-Joint series predictions and actual probabilities. Additionally, the DCA curves demonstrated that the Model-Joint series was clinically useful.

**Conclusion:**

Multiparametric MRI-based radiomics models can potentially predict tumor response after chemoradiotherapy in patients with postoperative residual gliomas, which may aid clinical decision making, especially to help patients initially predicted to be treatment-insensitive avoid the toxicity of chemoradiotherapy.

## Introduction

Glioma is the most common tumor of the brain and is associated with high rates of disability and death, which is part because the tumor lesion is difficult to completely remove surgically due to its invasive growth characteristics. Presently, according to the international guidelines for the treatment of neurological tumors, concurrent postoperative chemoradiotherapy is recommended for some grade 2, grade 3 and grade 4 glioma patients to treat residual tumor lesions. Radiotherapy and chemotherapy have a definite therapeutic effect on gliomas, but there are also some negative effects, such as hair loss, vomiting, decreased immunity and high cost. Clinical practices have shown that not all gliomas are sensitive to chemoradiation due to the heterogeneity of tumor tissues. Therefore, some patients not only are unable to benefit from the treatment but also unnecessarily suffer from the effects of chemoradiation. Therefore, identifying treatment-insensitive patients has become a critical area of research.

Previously, most studies predicting the response of tumors to radiotherapy and chemotherapy have been based on conventional computed tomography/magnetic resonance imaging (CT/MRI), functional MRI or positron emission tomography (PET), but the predictive performances have not been satisfactory. One of the important reasons is because the deep information within images cannot be comprehended with the naked eye. However, radiomics, an emerging method, can acquire the detailed characteristics from medical imaging data of the entire lesion in a high-throughput manner with computer technology (1–3). These extracted data can then be deeply mined and analyzed, correlated with the clinical or biological information of the disease, and finally used to construct prediction models.

Some studies evaluating tumor response with radiomics have been reported, but the research has mainly focused on colorectal cancer, nasopharyngeal cancer, breast cancer and lung cancer (4–7), showing improved predictive performance with radiomics. However, to the best of our knowledge, few studies predicting the curative effect of chemoradiotherapy for residual gliomas with a radiomics approach have been reported, but this area is very important for choosing treatment for glioma patients.

Thus, the purpose of this study was to extract high-throughput radiomics features from conventional multiparametric MR images, screen for features highly correlated with chemoradiation sensitivity, and establish logistic regression models based on the selected features to predict the treatment sensitivity of gliomas. Ultimately, the model was expected to accurately identify treatment-insensitive patients and thus aid clinicians in optimizing treatment regimens for such patients.

## Materials and Methods

### Patients

This is a retrospective study approved by our institution’s review board, and the requirement for informed consent was waived. In total, the images and pathological data of 231 consecutive patients were collected from three institutions from January 2015 to September 2020 (institution 1: The Second Affiliated Hospital of Nanchang University; institution 2: The First Affiliated Hospital of Gannan Medical College; and institution 3: Hsiang-ya Hospital). In total, 84 glioma patients with residual gliomas after surgery who were treated with concurrent chemotherapy and radiotherapy were enrolled in this study (institution 1, 57 patients; institution 2, 8 patients; and institution 3, 19 patients). The inclusion criteria were as follows: 1) tumors located above the cerebellar tentorium; 2) pathologically confirmed grade II-IV gliomas; 3) a definite residual tumor confirmed by conventional and advanced MRIs acquired within 72 hours after surgery, as well as clear surgical records of a residual lesion; 4) treatment with concurrent radiotherapy and chemotherapy after surgery within 3 weeks; and 5) available and complete preoperative and postoperative images, pathological data and clinical data. The exclusion criteria were as follows: 1) unclear presence of a residual tumor after surgery; 2) atypical postoperative treatment; and 3) missing or incomplete imaging or pathological data. All patients from institution 1 (57 patients), with a treatment-sensitive-to -treatment-insensitive ratio of 16:41, were defined as the training cohort. Patients from institution 2 and institution 3 (a total of 27 patients), with a treatment-sensitive-to-treatment-insensitive ratio of 9:18, were defined as the validation cohort.

### Image Acquisition

Preoperatively and postoperatively, conventional MR scans and contrast enhancement scans were acquired for the 84 glioma patients using different devices with similar scanning protocols. In institution 1, 57 patients underwent head MRI using a 1.5-T (Signa HDxt;GE Medical System, Inc, Waukesha, WI, USA) or 3.0-T device (Discovery 750; GE Healthcare, Milwaukee, WI, USA) with an 8-channel head coil. In institution 2, 8 patients underwent head MRI using 3.0-T device (Discovery 750; GE Healthcare, Milwaukee, WI, USA). In institution 3, 19 patients underwent head MRI using a 1.5-T (Avanto Magnetom; Siemens, Erlangen, Germany) or 3.0-T device (Discovery 750; GE Healthcare, Milwaukee, WI, USA). Preoperative MR images were taken within 2 weeks before the operation. Postoperative images were taken within 72 hours after surgery and 3 months after recurrent concurrent chemotherapy and radiotherapy. All of the scanning protocols and imaging parameters were similar among the different MR machines. Axial T1-weighted (T1-w), T2-weighted (T2-w) and axial contrast-enhanced T1-weighted (CET1-w) images in Digital Communications in Medicine (DICOM) format were selected for further analysis. Pathology results and clinical data were obtained from the Electronic Hospital Information System (EHIS).

### Image Normalization

To eliminate the heterogeneity among the MR scan parameters and devices, all images were resampled to 1*1*1 mm³ voxels, and their intensity range was normalized to 0 to 255 using the open-source 3D-Slicer 4.10.2 platform (https://www.softpedia.com/get/Science-CAD/3D- Slicer. shtml).

### Treatment and Response Evaluation

All 84 glioma patients from the three institutions began chemoradiotherapy within 3 weeks after surgery. Regarding, radiation range and dose, the gross tumor volume (GTV) included areas of abnormal enhancement and the lumen shown by postoperative MR CET1-w images and areas with abnormal signal on T2-w/FLAIR images. The clinical target volume (CTV) was defined as the GTV plus a margin of 1–2 cm. The radiotherapy dose was 60 Gy/30 fractions, avoiding any organs at risk. The chemotherapy regimen was implemented simultaneously and composed of temozolomide (75 mg/m^2^). The Response Assessment in Neuro Oncology (RANO) criteria (8) was currently widely used around the world to evaluate the effect of therapy on brain tumors and was also applied in this study to evaluate the treatment sensitivity of residual gliomas. The details of the RANO criteria were shown in the **Supplementary Material** (1). Partial remission and complete remission were defined by the presence of treatment-sensitive tumors that disappeared or decreased by ≥ 50% on CET1-w images. Stable disease and progressive disease were defined by the presence of treatment-insensitive tumors that increased or decreased by -25% ~ +25% on CET1-w images, lesions that increased by ≥ 25% and any new lesions. The therapeutic sensitivity of a tumor was defined by a combination of imaging findings and some clinical indexes. The approximate evaluation formula for tumor volume change was as follows: Tumor volume ≈ (Maximum long diameter) * (Vertical Maximum short diameter) * (Sum of all layer spacing and layer thickness). The first follow-up MRI was conducted within 72 hours postoperatively and applied as the baseline data.

### ROI Segmentation and Feature Extraction

Regions of interest (ROIs) were segmented on preoperative images (T1-w, T2-w, and CET1-w images) using the 3D-Slicer 4.10.2 platform, which was also used for feature extraction. All ROI boundaries in each tumor were delineated on every slice of the T1-w, T2-w and CET1-w images. Twenty random patients’ MR images were chosen and manually segmented by a radiologist with 10 years of experience in diagnosing central nervous system diseases (reader 1), and this process was repeated after 3 weeks to evaluate intraobserver reproducibility. The same segmentation procedure was conducted by another radiologist (reader 2) with 15 years of clinical experience to evaluate interobserver reproducibility.

Four types of radiomics features, shape, first-order, textural, and filter-based features (wavelet features), were extracted from the ROIs through the 3D-Slicer 4.10.2 platform. Additionally, four groups of features were extracted from the T1-w, T2-w, CET1-w and Joint series images.

Semantic features of the lesions, such as enhancement grade, cystic grade and edema grade were extracted from the conventional images. The enhancement, cystic and edema features were all graded into three levels, and the details were shown in **Table 1**.

**Table 1:** Clinical characteristics of the patients and semantic image features in the training and validation cohorts.

Characteristic	All patients	Training cohort	Validation cohort	P-value	Training and Validation cohort		P-value
	(n = 84)	(n = 57)	(n = 27)		Sensitive (n = 25)	Insensitive (n = 59)	
Age (years)	48.452 (12.619)	49.491 (11.386)	46.259 (14.891)	0.276	47.360 (12.312)	48.915 (12.823)	0.609
Gender:				0.840			0.535
male	48 (57.1%)	33 (57.9%)	15 (55.6%)		13 (52.0%)	35 (59.3%)	
female	36 (42.9%)	24 (42.1%)	12 (44.4%)		12 (48.0%)	24 (40.7%)	
Pathological grade:				0.262			0.330
grade 2	16 (19.0%)	12 (21.1%)	4 (14.8%)		7 (28.0%)	9 (15.3%)	
grade 3	19 (22.6%)	10 (17.5%)	9 (33.3%)		4 (16.0%)	15 (25.4%)	
grade 4	49 (58.3%)	35 (61.4%)	14 (51.9%)		14 (56.0%)	35 (59.3%)	
Enhancement grade:				0.605			0.474
grade 1	11 (13.1%)	8 (14.0%)	3 (11.1%)		5 (20.0%)	6 (10.2%)	
grade 2	40 (47.6%)	25 (43.9%)	15 (55.6%)		11 (44.0%)	29 (49.2%)	
grade 3	33 (39.3%)	24 (42.1%)	9 (33.3%)		9 (36.0%)	24 (40.7%)	
Cystic grade:				0.936			0.747
grade 1	20 (23.8%)	14 (24.6%)	6 (22.2%)		6 (24.0%)	14 (23.7%)	
grade 2	29 (34.5%)	20 (35.1%)	9 (33.3%)		10 (40.0%)	19 (32.2%)	
grade 3	35 (41.7%)	23 (40.4%)	12 (44.4%)		9 (36.0%)	26 (44.1%)	
Edema grade:				0.246			0.765
grade 1	10 (11.9%)	8 (14.0%)	2 (7.4%)		2 (8.0%)	8 (13.6%)	
grade 2	33 (39.3%)	19 (33.3%)	14 (51.9%)		10 (40.0%)	23 (39.0%)	
grade 3	41 (48.8%)	30 (52.6%)	11 (40.7%)		13 (52.0%)	28 (47.5%)	
Sensitive:				0.622			
yes	25 (29.8%)	16 (28.1%)	9 (33.3%)				
no	59 (70.2%)	41 (71.9%)	18 (66.7%)				

Continuous variables were presented as the mean (SD). Categorical variables were presented as absolute numbers (n) and proportions (%). Student’s t-test, χ2 test and Fisher’s exact test were used for comparisons of continuous variables and categorical variables, respectively.

Enhancement grade, according to visual enhancement:

grade 1 (mild enhancement); grade 2 (moderate enhancement); grade 3 (severe enhancement).

Cystic grade, according to the ratio of cystic volume to total lesion volume:

grade 1 (none); grade 2 (<50%); grade 3 (>50%).

Edema grade, according to the distance between the edge of the area of edema and lesion:

grade 1 (none); grade 2 (<2 cm); and grade 3 (>2 cm).

### Feature Selection, Radiomics Model Development, Model Performance Evaluation

The feature data from T1-w, T2-w, CET1-w and Joint series (T1-w+T2-w+CET1-w) were loaded into the GE IPMs platform. The synthetic minority oversampling technique (SMOTE) algorithm was used to balance the training datasets, and Z-score standardization was used for data normalization. After SMOTE, the training cohort contained 82 samples, with a treatment-sensitive -to -treatment-insensitive ratio of 41:41. To establish the radiomics signature, we used univariate analysis and multivariate analysis, including the Student’s t test, Rank sum test, Variance analysis, Correlation_analysis, Univariate _Logistic analysis and multivariate logistic analysis, to select feature sets from the normalized data. The detailed extraction process was showed in the **Supplementary Materials** (2–5).

R-4.0.3 (https://www.r-project.org/) and the R studio platform (https://rstudio.com/products/rstudio/download/#download) were used to develop the radiomics models. Features selected from the T1-w, T2-w, CET1-w, and Joint series were analyzed with multiple logistic regression to construct the radiomics models. Model validation was conducted in the validation cohort. Calibration plots and receiver operating characteristic (ROC) curves were generated to evaluate model performance. Decision curve analysis (DCA) was performed to evaluate the clinical utility of the models.

### Statistical Analysis

Statistical analysis was performed with IBM SPSS Statistics 25 and R software (version 4.0.3). Student’s t test, Chi-square tests and Fisher’s exact tests were used to compare the clinical characteristics, image and pathology data of the training and validation cohorts. The Delong test was used to compare differences in the ROC curves among various models. The intraclass correlation coefficient (ICC) was applied to assess the stability of each extracted radiomics feature. ICCs were calculated through the ‘irr’ package. Multivariate binary logistic regression was performed with the ‘glmnet’ package. The ‘pROC’, ‘rms’ and ‘rmda’ packages were used to obtain ROC curves, calibration curves and DCA curves, respectively. A two-tailed P-value < 0.05 was considered statistically significant.

## Results

### Patient Characteristics

No significant differences in patient characteristics were observed between the training and validation cohorts (age, sex, sensitivity to treatment, pathological grade, enhancement grade, cystic grade, and edema grade, **Table 1**, all P > 0.05), indicating that it was reasonable to use external data from institutions 2 and 3 for validation.

### Intraobserver and Interobserver Reproducibility of the Radiomics Features

A total of 2553 (851×3) radiomics features were extracted from the ROIs of the T1-w, T2-w and CET1-w images, respectively, including 18 first-order features,14 shape features, and 75 texture features [gray level dependence matrix (GLDM, 14), gray-level co-occurrence matrix (GLCM, 24), gray-level run length matrix (GLRLM, 16), gray-level size zone matrix (GLSZM, 16) and neighborhood gray-tone difference matrix (NGTDM, 5)], and 744 wavelet features [(18 + 14+24+16+16+5)*8]. Features with intraobserver and interobserver ICCs <0.75 were discarded. Therefore, 467 features from the T1-w images, 380 features from the T2-w images, and 490 features from the CET1-w images were obtained.

### Radiomics Signature and Model Construction

**Figure 1:**
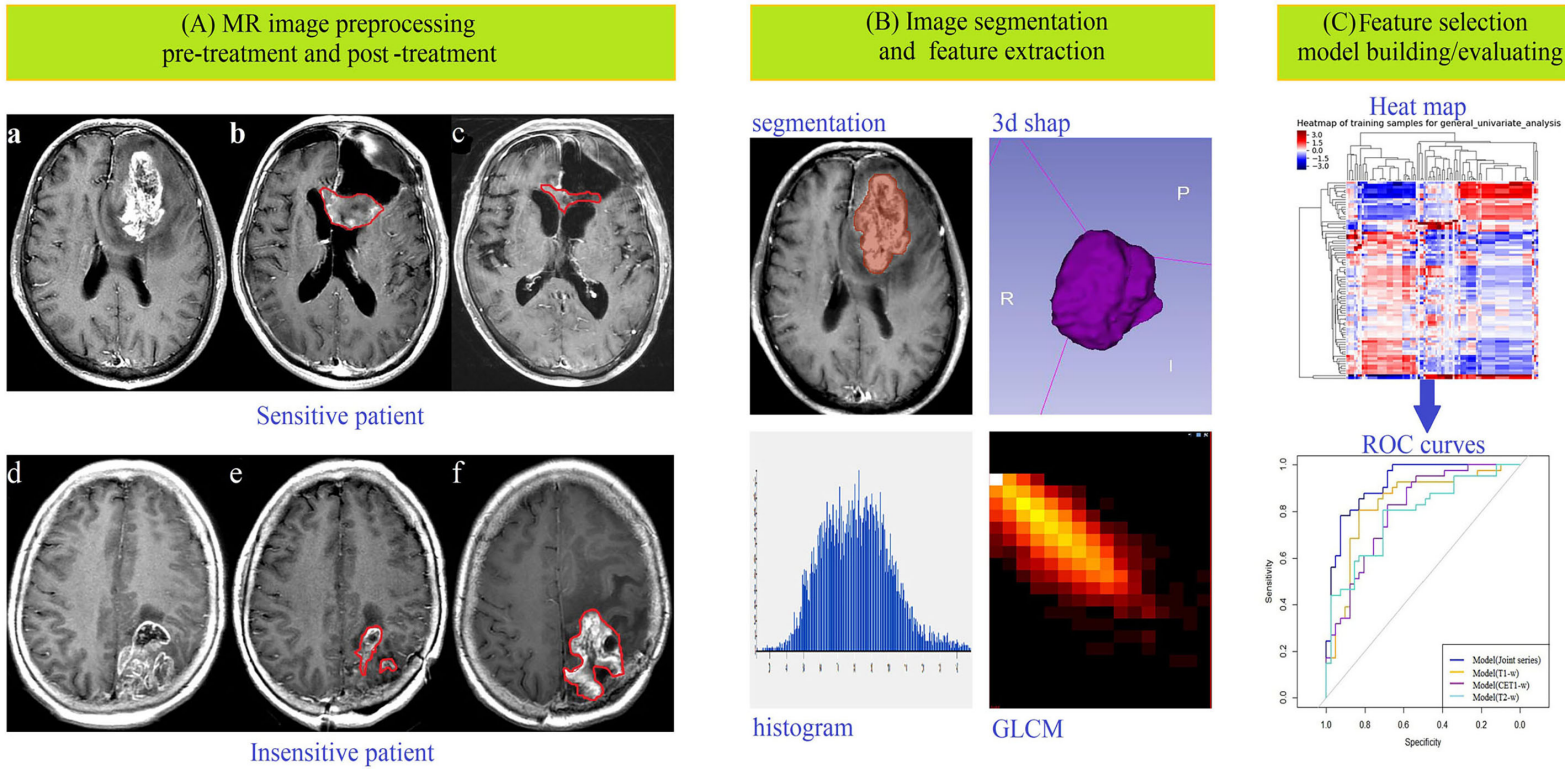
Data acquisition and analysis workflow. All patients were divided into treatment-sensitive and treatment-insensitive groups. **(A)** Pictures a and d showed the original images before treatment, b and e showed the postoperative images acquired within 24–72 hours, and c and f showed the postoperative images acquired after approximately 3 months of follow-up. **(B)** ROIs were defined, and feature extraction, including for first-order, shape-, high-order texture-, and filter-based features, was performed. **(C)** Feature selection, model building and model evaluation were used to predict the response of glioma patients to chemoradiation by multiple logistic regression analysis.

After univariate analysis with or without multivariate analysis, several features highly correlated with sensitivity to chemoradiation were identified (6 features from the T1-w images, 7 features from the T2-w images, 6 features from the CET1-w images, and 5 features from the Joint series images). The detailed screening procedures were showed in the **Supplementary Materials** (2–5). The data acquisition and analysis workflow was showed in **Figure 1**. These final features were introduced to build four radiomics models through multivariate binary logistic regression:

Model(T1-w)=-0.1262+(0.9157*wavelet-LLL_glcm_Idmn)-(0.1732*wavelet-LHL_glcm_Imc2)
+(0.8470*original_firstorder_Skewness)-(0.3914*wavelet-HLL_glcm_DifferenceAverage)
+(0.5724*wavelet-LHH_firstorder_Median)+(0.4312*wavelet-LHH_glcm_SumEntropy)
Model(T2-w)=0.0267-(0.2443*wavelet-LHL_gldm_LargeDependenceEmphasis)-(0.6148* original_glrlm_LongRunHighGrayLevelEmphasis)+(0.6830*wavelet-HHL_glcm_SumEntropy)
-(0.0707*original_glcm_Idn)+(0.7691*wavelet-HHL_glcm_Contrast)+(0.1583*wavelet-LHH
_firstorder_Maximum) -(0.1271*wavelet-LLL_glcm_Imc1)
Model(CET1-w)=-0.0983+(0.1927*wavelet-LHL_glrlm_RunVariance)-(0.9772*wavelet-LHH _glcm_Idmn)+(0.3411*wavelet-HLH_glszm_HighGrayLevelZoneEmphasis)-(0.8397*wavelet
-LHH_firstorder_Kurtosis)+(0.5278*wavelet-HLH_glszm_SmallAreaHighGrayLevelEmphasis)
-(0.5460*wavelet-HHL_firstorder_Kurtosis)
Model(Joint series)=-0.7185-(2.0591*T1_wavelet-LLL_glszm_GrayLevelNon
UniformityNormalized)-(1.9826*CET1_wavelet-LHH_glcm_Idmn)-(2.5979*CET1_wavelet
-LHH_firstorder_Kurtosis)+(1.2404*T1_wavelet-LLH_firstorder_Kurtosis)-(1.2912*CET1
_wavelet-LLH_firstorder_Median)

### Model Performance Evaluation

**Figure 2:**
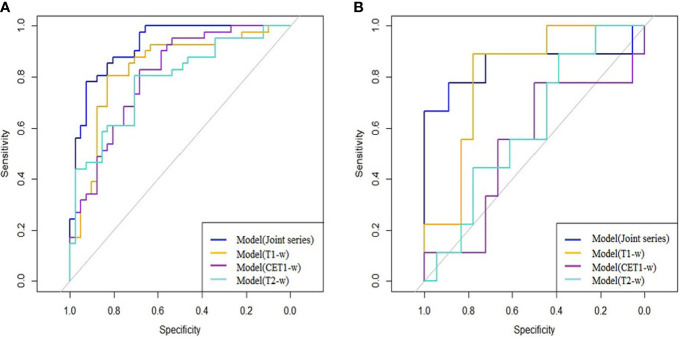
**(A, B)** showed the ROC curves of the four prediction models in the training and validation cohorts: the blue curve represented the Model(Joint series), the orange curve represented Model(T1-w), the purple curve represented Model(CET1-w), and the turquoise curve represented model(T2-w).

**Figure 3:**
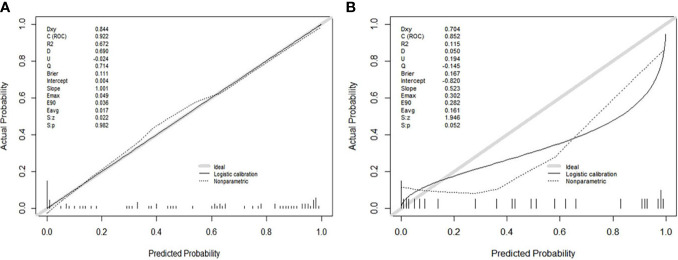
**(A, B)** showed the calibration curves of the Model(Joint series) in the training cohort and validation cohort. The y-axis represented the actual probability of treatment-sensitive patients. The x-axis represented the predicted probability of treatment-insensitive patients. The diagonal gray line represented a perfect prediction by an ideal model. The black solid line represented the prediction performance of the Model(Joint series), and the closer the black line was to the gray line, the better the prediction performance of the model.

**Figure 4:**
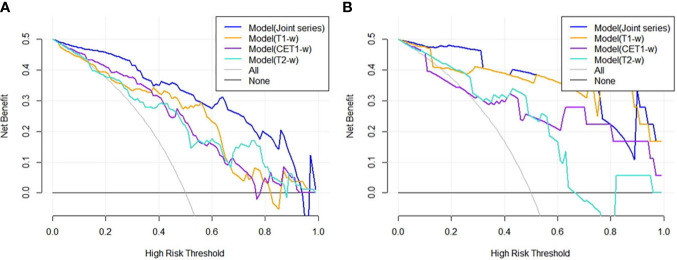
**(A, B)** showed the DCA results for four models in the training cohort and validation cohort. The blue curve was for the Model(Joint series), the orange curve was for Model(T1-w), the purple curve was for Model(CET1-w), and the turquoise curve was for Model(T2-w). The x- and y-axes indicated the high-risk threshold and net benefit, respectively. The gray curve represented the assumption that all patients were sensitive to treatment; the black line represented the assumption that all patients were insensitive to treatment.

The ROC curves (**Figure 2**) showed the Model-Joint series and Model-T1 had better performances, with areas under the ROC curves (AUCs) of 0.923 and 0.835 in the training cohort and 0.852 and 0.809 in the validation cohort, whereas Model-CET1 and Model-T2 had poor performances with low AUCs in the validation cohorts. Model-T1 had the best sensitivity, specificity and accuracy compared to the other models. The detailed performance results of the different models were listed in **Table 2**. The Delong tests did not find significant differences between the ROC curves of the Model-Joint series and Model-T1 (p values of 0.113 and 0.738 in the training and validation sets, respectively). However, the differences between the Model-Joint series and Model-CET1-w and between the Model-Joint series and Model-T2 were significant in the training sets, with p values of 0.014 and 0.012, respectively. Nonsignificant unreliability U test (P=0.982 and 0.052) and Hosmer-Lemeshow test results (P=0.7303 and 0.9084) showed good calibration in the training cohort and validation cohort regarding Model-Joint series. The calibration curves were showed in **Figure 3**. The DCA curves indicated that the Model-Joint series had the most clinical utility, as showed in **Figure 4**.

**Table 2:** The performances of the four logistic regression models in predicting sensitivity to treatment in the training and validation cohorts.

Modality	Features screening	Remainedfeatures	Cohorts	AUC (95%CI)	Sen	Spe	Acc
Model-Joint series	univariate analysis**+** multivariate analysis	5	training	0.923 (0.866-0.979)	0.829	0.829	0.829
			validation	0.852 (0.644-1.000)	0.778	0.722	0.741
Model-T1	univariate analysis	6	training	0.835 (0.743-0.927)	0.805	0.756	0.780
			validation	0.809 (0.638-0.979)	0.889	0.778	0.815
Model-CET1	univariate analysis	6	training	0.805 (0.712-0.899)	0.780	0.683	0.732
			validation	0.537 (0.284-0.790)	0.778	0.389	0.519
Model-T2	univariate analysis	7	training	0.784 (0.685-0.883)	0.732	0.707	0.720
			validation	0.605 (0.381-0.829)	0.444	0.556	0.519

AUC, area under the curve; Sen, sensitivity; Spe, specificity; Acc, accuracy.

Univariate analysis included ‘General_Univariate_analysis’ (Student’s t test or Rank sum test), ‘Variance’, ‘Correlation_xx’ and ‘Univariate _Logistic’analysis.

The multivariate analysis used in this study was ‘MultiVariate_Logistic’ analysis.

## Discussion

In our study, four MRI-based radiomics logistic regression models were established, derived from T1-w, T2-w, CET1-w and Joint series. Model-Joint series and Model-T1 yielded better predictivity in determining whether glioma patients were sensitive to chemoradiation. Hence, MRI-based radiomics models may help clinicians identify treatment-sensitive and treatment-insensitive patients to tailor treatment to each individual.

Radiotherapy and chemotherapy are important adjuvant treatments for patients with residual tumors postoperatively. Multiparameter MRI, a noninvasive and repeatable examination method, has been widely used to evaluate the efficacy of chemoradiotherapy for tumors. However, there are few studies predicting the curative effect of chemoradiotherapy for residual gliomas before treatment, which is crucial for choosing the right therapeutic schedule. We know that not all glioma patients are susceptible to chemoradiotherapy. Therefore, if we can detect poor responders early, many unnecessary injuries and side effects can be avoided. Additionally, alternative treatments can be chosen earlier, such as targeted therapy (9–12), immunotherapy (13–16), interstitial brachytherapy (17, 18), or even sonodynamic therapy based on ultrasound stimulation and a sonosensitizer (19). In our study, we used radiomics models to predict the sensitivity of residual gliomas to chemoradiotherapy and compared the histopathological grade and conventional MR semantic features between treatment-sensitive and treatment-insensitive patients. The results showed no significant difference in the histopathological grade of gliomas between the treatment-sensitive and treatment-insensitive groups. This may be because the histological grade was determined by the degree of differentiation of a small specimen, which cannot represent the whole tumor, whereas radiomics features provide the tumor’s overall biological information (20, 21). The pretreatment conventional MR semantic features of the tumor lesions, including enhancement, cystic and edema grades, also showed no difference between the two groups. Therefore, conventional MR semantic features may not predict the sensitivity of the tumor to chemoradiation.

Radiomics, an emerging and noninvasive research method, is used to extract high-throughput imaging features that cannot be recognized by the human eye and to evaluate and quantify biological information such as tumor heterogeneity, tumor cell growth and the surrounding microenvironment (22, 23). According to previous studies on locally advanced rectal cancer, radiomics features, including skewness, entropy and several GLCM parameters, extracted from MRI have can be highly significant predictors for the response to neoadjuvant chemoradiotherapy (24–27). In our present work, four groups of radiomics features were selected to establish predictive models, including first-order features, shape-based features, textural features, and wavelet features. The wavelet features were extracted by a three-dimensional discrete wavelet transform using high-frequency and low-frequency filters, which can accurately obtain the detailed features of the images. In the four models (**Table 3**), the wavelet_firstorder_Kurtosis feature appeared four times, indicating that kurtosis was closely associated with chemoradiotherapy in gliomas; this feature described the overall distribution curve of voxel intensity in the ROI with a flat or sharp peak. However, this result was different from that of Dinapoli N et al. (26), who conducted a study based on an intensity histogram signature to predict the probability of achieving a pathologic complete response using a Laplacian of Gaussian (LoG) filter. We inferred that there may be two reasons for the different results: one was the different types of tumors studied, and the other was the different feature extraction methods. The wavelet_glcm_Idmn feature was the second most repeated feature in all models. GLCM described the joint gray-level distribution of any two pixels in the ROI with some spatial positioning information, and the gray-level distribution can be mined with repetitive regularity. Idmn measured the local variations in the image texture and reflected the homogeneity of the texture. The more uniform the local image was, the stronger the texture regularity, and the larger the Idmn value. The Wavelet_firstorder_Median, wavelet_glcm_Imc and wavelet_glcm _SumEntropy features were repeated twice in the models. Median represented the median gray level intensity within the ROI, which was likely related to chemotherapeutic efficacy according the Ng SH’ study (28). Imc assessed the correlation of the probability distributions, quantifying the complexity of the texture. SumEntropy was the sum of the neighborhood intensity value differences. In the Model-Joint series, the Idmn and median values from the CET1-w scans were both inversely proportional to the sensitivity of glioma to chemoradiation. This meaned that the more heterogeneous the image texture, the smaller the median gray value, and the more sensitive the tumors were to treatment. In our four models, no shape-based features appeared, suggesting that tumor morphology was not closely related to sensitivity to treatment. Additionally, it was noteworthy that no feature was selected from the T2-w images to construct the Model-Joint series. We found most of the features selected from T1-w, T2-w and CET1-w images were texture features, which reflected the internal distribution law of image pixels. Usually, T2-w was significantly superior to T1-w in depicting lesions, but it may focus more on differences in signal strength. However, in analyzing the deep texture structure of the image, T1-w seemed to perform better than T2-w based on machine vision. In future studies, we will focus on this problem and further verify it.

**Table 3:** Four groups of radiomics features extracted from MR images were showed.

**Model(T1-w)**	**wavelet-LLL**	**wavelet-LHL**	**Original**	**wavelet-HLL**	**wavelet-LHH**	**wavelet-LHH**	
	**_glcm_Idmn**	**_glcm_Imc2**	**_firstorder_Skewness**	**glcm_DifferenceAverage**	**_firstorder_Median**	**_glcm_SumEntropy**	
**Model(T2-w)**	**wavelet-LHL_gldm**	**Original_glrlm**	**wavelet-HHL**	**Original**	**wavelet-HHL**	**wavelet-LHH**	**wavelet-LLL**
	**_LargeDependenceEmphasis**	**_LongRunHighGrayLevelEmphasis**	**_glcm_SumEntropy**	**_glcm_Idn**	**_glcm_Contrast**	**_firstorder_Maximum**	**_glcm_Imc1**
**Model(CET1-w)**	**wavelet-LHL**	**wavelet-LHH**	**wavelet-HLH_glszm**	**wavelet-LHH**	**wavelet-HLH_glszm**	**wavelet-HHL**	
	**_Glrlm_RunVariance**	**_glcm_Idmn**	**_HighGrayLevelZoneEmphasis**	**_firstorder_Kurtosis**	**_SmallAreaHighGrayLevelEmphasis**	**_firstorder_Kurtosis**	
**Model(Joint series)**	**T1_wavelet-LLL_glszm**	**CET1_wavelet-LHH**	**CET1_wavelet-LHH**	**T1_wavelet-LLH**	**CET1_wavelet-LLH**		
	**_GrayLevelNonUniformity Normalized**	**_glcm_Idmn**	**_firstorder_Kurtosis**	**_firstorder_Kurtosis**	**_firstorder_Median**		

Features of the same class appeared in the same color to distinguish them from other features.

Among all four models, the Model-Joint series had the best prediction performance for the sensitivity of residual gliomas to treatment, followed by Model-T1, but the Delong test showed that the difference in AUC was not significant between these two models. The reason for this may be because the sample size was not large enough. However, the Delong tests for the comparisons between the Model-Joint series and Model-CET1(p=0.014) and between the Model-Joint series and Model-T2(p=0.012) in the training cohort showed significant differences. In addition, more samples and more complex deep learning algorithms need to be used to further improve the sensitivity, specificity and accuracy of the Model-Joint series in the validation cohort. Nonetheless, one strength of this study was that MRI was used to noninvasively predict both radiotherapy and chemotherapy outcomes for gliomas before treatment beginning, which was worthy of further study in the future.

There are several limitations to this work. First, although the RANO criteria are the current standard for evaluating clinical therapeutic effects in tumors, there are still a few cases that are difficult to classify. Therefore, we asked a chief physician with 30 years of experience to confirm the results. Second, it is more reasonable to enlarge sample in future to compare differences in clinical, pathological, and semantic features between treatment-sensitive and treatment-insensitive patients. In addition, many previous studies have shown that genotype, such as IDH mutation, 1p19q codeletion and MGMT promoter methylation status, has a great correlation with therapeutic effect and tumor prognosis (29, 30). Due to the absence of genetic tests in some patients, genetic phenotype was not included as a predictor of treatment response in this study. However, in the future, it is necessary to conduct prospective studies that integrate additional genetic information into the predictive models.

In conclusion, we explored the potential role of radiomics-based models derived from preoperative multiparametric MRI in predicting the response to concurrent radiotherapy and chemotherapy in residual glioma patients. These models may help clinicians personalize patient treatment and help treatment-insensitive patients avoid unnecessary injuries and side effects from chemoradiotherapy.

## Data Availability Statement

The original contributions presented in the study are included in the article/**Supplementary Material**. Further inquiries can be directed to the corresponding author.

## Ethics Statement

The studies involving human participants were reviewed and approved by The Second Affiliated Hospital of Nanchang University Medical Research Ethics Committee. Written informed consent for participation was not required for this study in accordance with the national legislation and the institutional requirements.

## Author Contributions

ZZ and KH carried out the research and drafted the original manuscript. ZZ, YZ, and DW collected the data. ZZ, ZW, and JZ analyzed the data and built the prediction models. LZ and YY evaluated the efficacy of tumor treatment. TG and XX revised the manuscript. All authors contributed to the article and approved the submitted version.

## Funding

This study was supported by the National Natural Science Foundation of China (NO. 82060557), the Key Research & Development Program of Jiangxi Province, China (NO. 20171ACG70002), Natural Science Foundation of Hunan province, China (NO. 2018JJ2271), as well as Science and technology planning project of Jiangxi Provincial Health and Family Planning Commission (No. 20195247).

## Conflict of Interest

The authors declare that the research was conducted in the absence of any commercial or financial relationships that could be construed as a potential conflict of interest.

The reviewer JL declared a shared parent affiliation with one of the authors, YZ, to the handling editor at time of review.

## Publisher’s Note

All claims expressed in this article are solely those of the authors and do not necessarily represent those of their affiliated organizations, or those of the publisher, the editors and the reviewers. Any product that may be evaluated in this article, or claim that may be made by its manufacturer, is not guaranteed or endorsed by the publisher.
