# Supplementary material for: Multiparametric MRI Radiomics for the Early Prediction of Response to Chemoradiotherapy in Patients With Postoperative Residual Gliomas: An Initial Study
Source: Front Oncol. 2021 Nov 18;11:779202. doi: 10.3389/fonc.2021.779202 (PMC8636428; doi:10.3389/fonc.2021.779202)
Supplement: Supplementary file 1 [file DataSheet_1.doc]

Clinical efficacy evaluation method for neurotumor (RANO Standard).

| Criterion | Complete remission(CR) | Partial remission(PR) | Stable disease(SD) | Progress disease(PD) |
| --- | --- | --- | --- | --- |
| MR CET1-w | Not seen(tumor lesion) | Decrease ≥ 50% | Increase or decrease in the range of -25%~+25% | Increase ≥ 25%* |
| MR T2-w/FLAIR | Stable or diminished | Stable or diminished | Stable or diminished | Increase* |
| New lesion | None | None | None | Present* |
| Corticosteroids | None | Stable or diminished | Stable or diminished | Not apply |
| Clinical status | Stable or improve | Stable or improve | Stable or improve | Deteriorate* |
| Conditions for evaluation | All | All | All | Any |

Abbreviation: MR, magnetic resonance; CE, contrast enhanced; FLAIR, Fluid-attenuated inversion recovery.

*Progress disease is determined by any one project.
