# Supplementary material for: Multiparametric MRI Radiomics for the Early Prediction of Response to Chemoradiotherapy in Patients With Postoperative Residual Gliomas: An Initial Study
Source: Front Oncol. 2021 Nov 18;11:779202. doi: 10.3389/fonc.2021.779202 (PMC8636428; doi:10.3389/fonc.2021.779202)

**Statistical Report**

Author: DESKTOP-LLEB9HD

Data: 'train_Joint series.csv'

# Summary Report

# 1. Data: C:/Users/Dell-EFY/Desktop/data/train_Joint series.csv

# 2. Random seed: 15

# 3. Seperative rate: 1.0

Seperated report:

|  | Sum | Pos | Neg |
| --- | --- | --- | --- |
| data | 82 | 41 | 41 |
| train | 82 | 41 | 41 |
| test | 0 | 0 | 0 |

# 4. The method for standardizing the data: Standardization

# 5. The method for selecting features: General_Univariate_analysis

parameters setted: {'P value for threshold in': 0.05}
num of remained features: 263
remained features:
[['T1cHighGrayLevelRunEmphasis.8']
 ['t2Mean.8']
 ['T1cSkewness.8']
 ['t1Id.4']
 ['t1Autocorrelation.4']
 ['t2InterquartileRange.6']
 ['t2MeanAbsoluteDeviation']
 ['t1InterquartileRange']
 ['t2DifferenceAverage.7']
 ['T1cKurtosis.6']
 ['t2Mean.7']
 ['t2RootMeanSquared.6']
 ['T1cVariance.6']
 ['t1InverseVariance.8']
 ['t1Id.6']
 ['t2MeanAbsoluteDeviation.5']
 ['t2Idm.7']
 ['t1MCC.6']
 ['t2Energy.5']
 ['t1SmallDependenceHighGrayLevelEmphasis.1']
 ['t290Percentile']
 ['T1c10Percentile.6']
 ['T1cRunVariance.1']
 ['T1cInterquartileRange.3']
 ['t2LargeDependenceEmphasis.8']
 ['t2TotalEnergy.5']
 ['t1Complexity.8']
 ['t1DifferenceEntropy.6']
 ['t1MaximumProbability.6']
 ['t1Contrast.8']
 ['T1cEnergy.2']
 ['t2Median.6']
 ['t2HighGrayLevelZoneEmphasis']
 ['t1Variance.7']
 ['t2Uniformity.6']
 ['t2DifferenceVariance.8']
 ['t1ClusterTendency.6']
 ['t1Contrast.16']
 ['T1cSkewness.7']
 ['T1cTotalEnergy.2']
 ['T1cJointAverage.8']
 ['t190Percentile']
 ['t1GrayLevelVariance.23']
 ['t2Variance.5']
 ['t1DifferenceAverage.8']
 ['t1Idm.6']
 ['T1cMaximum.5']
 ['T1cRootMeanSquared.5']
 ['t1Complexity.4']
 ['t2Contrast.14']
 ['t2RunLengthNonUniformityNormalized.8']
 ['t2RunPercentage.8']
 ['t1RunPercentage.8']
 ['t1InverseVariance.4']
 ['T1cRobustMeanAbsoluteDeviation.2']
 ['t2Id.1']
 ['t2Variance']
 ['t1LongRunLowGrayLevelEmphasis.1']
 ['T1c10Percentile.1']
 ['T1c10Percentile.5']
 ['T1cUniformity.3']
 ['t290Percentile.1']
 ['t1Contrast']
 ['t2RootMeanSquared']
 ['t1Correlation']
 ['t2SmallDependenceHighGrayLevelEmphasis.7']
 ['T1cSmallDependenceHighGrayLevelEmphasis.2']
 ['t1LowGrayLevelRunEmphasis.1']
 ['T1cComplexity.2']
 ['T1c90Percentile.1']
 ['t2RobustMeanAbsoluteDeviation.6']
 ['t2Energy.6']
 ['t1InverseVariance.7']
 ['t2Id.8']
 ['t1Idm']
 ['t2RootMeanSquared.5']
 ['t2Id.7']
 ['T1cInterquartileRange.6']
 ['T1c90Percentile.6']
 ['t1LargeDependenceEmphasis.8']
 ['t1Correlation.6']
 ['t2RootMeanSquared.2']
 ['t1GrayLevelVariance.22']
 ['t2DifferenceEntropy.8']
 ['T1cContrast.5']
 ['t1Uniformity']
 ['t1Idm.8']
 ['t290Percentile.4']
 ['t1Idmn.4']
 ['t1DifferenceAverage.4']
 ['t1Idm.4']
 ['t1DifferenceVariance.8']
 ['t1Idm.7']
 ['t2DependenceEntropy.8']
 ['t1GrayLevelNonUniformityNormalized.15']
 ['t1ClusterProminence.8']
 ['t1Strength.6']
 ['t1Skewness.8']
 ['t2RootMeanSquared.7']
 ['t1Correlation.4']
 ['t210Percentile.8']
 ['t2Idn.8']
 ['T1cJointEnergy.1']
 ['t2MeanAbsoluteDeviation.6']
 ['t1GrayLevelVariance.21']
 ['t1JointEnergy.6']
 ['t2RootMeanSquared.8']
 ['t290Percentile.7']
 ['t1JointAverage.1']
 ['t210Percentile.1']
 ['T1cRobustMeanAbsoluteDeviation.6']
 ['t290Percentile.2']
 ['t1Kurtosis.3']
 ['t1Contrast.14']
 ['T1cSmallDependenceLowGrayLevelEmphasis.8']
 ['t2MeanAbsoluteDeviation.2']
 ['T1cKurtosis.2']
 ['T1cMeanAbsoluteDeviation.6']
 ['t1Maximum.7']
 ['t2SumSquares.6']
 ['T1cGrayLevelVariance.9']
 ['t2DifferenceAverage.8']
 ['t2ClusterProminence.7']
 ['t1Strength.7']
 ['t2InterquartileRange.1']
 ['t2InverseVariance.7']
 ['t1ClusterTendency.4']
 ['t210Percentile.4']
 ['t1Complexity.7']
 ['t2Contrast.16']
 ['t2Median.7']
 ['T1cMeanAbsoluteDeviation.5']
 ['T1cDependenceVariance.3']
 ['t1Range.7']
 ['t1InverseVariance']
 ['T1c90Percentile.5']
 ['T1c10Percentile.4']
 ['T1cAutocorrelation.8']
 ['T1cKurtosis.5']
 ['t2Maximum.7']
 ['t1Range.8']
 ['T1cMeanAbsoluteDeviation.4']
 ['t2Variance.4']
 ['t1Imc2.6']
 ['t2MeanAbsoluteDeviation.4']
 ['t1DifferenceEntropy.7']
 ['T1cInterquartileRange.4']
 ['t210Percentile.5']
 ['t2MeanAbsoluteDeviation.1']
 ['T1cIdmn.2']
 ['t1DifferenceAverage.6']
 ['T1c90Percentile.4']
 ['t290Percentile.5']
 ['T1cRobustMeanAbsoluteDeviation.4']
 ['t1HighGrayLevelEmphasis.1']
 ['T1cRootMeanSquared.6']
 ['t2ClusterShade.6']
 ['T1cVariance.5']
 ['t2Maximum.1']
 ['t1Autocorrelation.1']
 ['t2TotalEnergy.6']
 ['t2DifferenceAverage.1']
 ['t2RobustMeanAbsoluteDeviation.1']
 ['t2Variance.1']
 ['t210Percentile.6']
 ['t2InverseVariance.8']
 ['t2LowGrayLevelZoneEmphasis.1']
 ['t2InterquartileRange']
 ['T1cRobustMeanAbsoluteDeviation.5']
 ['t1Contrast.9']
 ['T1cInterquartileRange.5']
 ['T1cInterquartileRange.2']
 ['t2ShortRunHighGrayLevelEmphasis.6']
 ['t1Id.7']
 ['t2RobustMeanAbsoluteDeviation']
 ['t1LowGrayLevelEmphasis.1']
 ['t1DifferenceVariance.7']
 ['t290Percentile.8']
 ['T1cRobustMeanAbsoluteDeviation']
 ['T1cHighGrayLevelEmphasis.8']
 ['T1cIdn.2']
 ['t1Variance.8']
 ['T1cMedian.3']
 ['t2Range.7']
 ['t2ShortRunEmphasis.8']
 ['t210Percentile.2']
 ['T1cSmallAreaHighGrayLevelEmphasis.4']
 ['t1LargeDependenceHighGrayLevelEmphasis.1']
 ['t2Maximum.8']
 ['t2DependenceNonUniformityNormalized.8']
 ['t2ClusterProminence.8']
 ['t1ClusterProminence.7']
 ['t2RootMeanSquared.1']
 ['t1Maximum.1']
 ['t2SmallDependenceHighGrayLevelEmphasis.8']
 ['t2JointEntropy.7']
 ['t1Idn.4']
 ['T1cContrast.2']
 ['t2Variance.6']
 ['t1Uniformity.1']
 ['T1cInterquartileRange']
 ['t1DifferenceVariance.6']
 ['T1c90Percentile.2']
 ['t2Contrast.17']
 ['t2HighGrayLevelRunEmphasis.6']
 ['t210Percentile']
 ['t1Imc1.6']
 ['t2Range.8']
 ['t1Maximum.8']
 ['t2Idm.8']
 ['T1cRootMeanSquared.2']
 ['t2LargeDependenceEmphasis.7']
 ['t1SumAverage.1']
 ['t2RootMeanSquared.4']
 ['t1GrayLevelVariance.25']
 ['t1ClusterProminence.4']
 ['t1GrayLevelNonUniformityNormalized.16']
 ['t2LongRunEmphasis.8']
 ['T1c10Percentile.2']
 ['T1cSkewness']
 ['t1Contrast.12']
 ['t2Median.8']
 ['t2Variance.2']
 ['t1Skewness.7']
 ['t290Percentile.6']
 ['t1RobustMeanAbsoluteDeviation']
 ['t1HighGrayLevelRunEmphasis.1']
 ['T1cBusyness.6']
 ['t1ClusterProminence.6']
 ['T1cKurtosis.4']
 ['t1DependenceNonUniformityNormalized.7']
 ['t2Idm.1']
 ['t1ClusterShade.7']
 ['T1cLargeDependenceHighGrayLevelEmphasis.8']
 ['t1MeanAbsoluteDeviation.7']
 ['t1DifferenceAverage.7']
 ['t1DependenceNonUniformityNormalized.8']
 ['T1cMaximumProbability.1']
 ['T1cMeanAbsoluteDeviation.2']
 ['t1InverseVariance.6']
 ['t1MeanAbsoluteDeviation.8']
 ['T1cRobustMeanAbsoluteDeviation.1']
 ['T1cVariance.2']
 ['t2GrayLevelVariance.19']
 ['t2RunPercentage.7']
 ['t2Complexity.8']
 ['t1Id']
 ['T1cSumAverage.8']
 ['t1Median.6']
 ['t1HighGrayLevelZoneEmphasis.7']
 ['T1cMeanAbsoluteDeviation.1']
 ['t2ClusterShade.7']
 ['t1DifferenceEntropy.8']
 ['t2GrayLevelNonUniformityNormalized.12']
 ['T1cInterquartileRange.1']
 ['T1cTotalEnergy.5']
 ['t1Id.8']
 ['t210Percentile.7']
 ['t1LongRunHighGrayLevelEmphasis.7']
 ['T1cEnergy.5']
 ['T1cKurtosis']
 ['t1SumEntropy.6']
 ['t2Complexity.7']]

Heatmap of the model in the training samples:


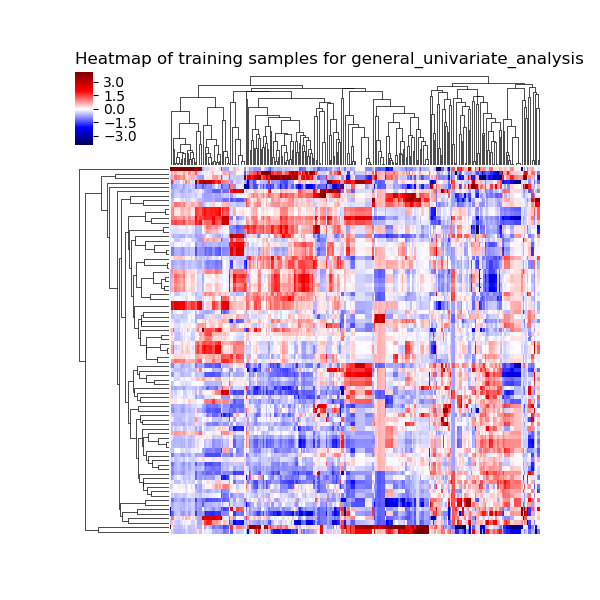


# 6. The method for selecting features: Variance

parameters setted: {'threshold': 1.0}
num of remained features: 98
remained features:
[['T1cHighGrayLevelRunEmphasis.8']
 ['t2DifferenceAverage.7']
 ['t2Mean.7']
 ['t1InverseVariance.8']
 ['t1Id.6']
 ['t2MeanAbsoluteDeviation.5']
 ['t2Energy.5']
 ['t290Percentile']
 ['T1cRunVariance.1']
 ['t2TotalEnergy.5']
 ['t1DifferenceEntropy.6']
 ['t2HighGrayLevelZoneEmphasis']
 ['t2Uniformity.6']
 ['t2RunPercentage.8']
 ['t2Variance']
 ['T1c10Percentile.1']
 ['T1c10Percentile.5']
 ['t290Percentile.1']
 ['t2SmallDependenceHighGrayLevelEmphasis.7']
 ['T1cSmallDependenceHighGrayLevelEmphasis.2']
 ['T1cComplexity.2']
 ['t2Energy.6']
 ['t1InverseVariance.7']
 ['T1cInterquartileRange.6']
 ['t1GrayLevelVariance.22']
 ['t2DifferenceEntropy.8']
 ['T1cContrast.5']
 ['t1Uniformity']
 ['t1Idmn.4']
 ['t2DependenceEntropy.8']
 ['t1GrayLevelNonUniformityNormalized.15']
 ['t2RootMeanSquared.7']
 ['t1Correlation.4']
 ['t2MeanAbsoluteDeviation.6']
 ['t2RootMeanSquared.8']
 ['t290Percentile.7']
 ['t1Kurtosis.3']
 ['t2MeanAbsoluteDeviation.2']
 ['T1cKurtosis.2']
 ['t1Maximum.7']
 ['T1cGrayLevelVariance.9']
 ['t1Strength.7']
 ['t2InterquartileRange.1']
 ['t1ClusterTendency.4']
 ['t1Complexity.7']
 ['t2Contrast.16']
 ['t2Median.7']
 ['T1cMeanAbsoluteDeviation.5']
 ['t1InverseVariance']
 ['T1c90Percentile.5']
 ['T1c10Percentile.4']
 ['T1cKurtosis.5']
 ['t1Range.8']
 ['t2Variance.4']
 ['t1Imc2.6']
 ['t2MeanAbsoluteDeviation.4']
 ['t1DifferenceEntropy.7']
 ['T1cInterquartileRange.4']
 ['t2MeanAbsoluteDeviation.1']
 ['T1cIdmn.2']
 ['T1c90Percentile.4']
 ['t290Percentile.5']
 ['t1Autocorrelation.1']
 ['t2TotalEnergy.6']
 ['t2DifferenceAverage.1']
 ['t2Variance.1']
 ['t210Percentile.6']
 ['t2LowGrayLevelZoneEmphasis.1']
 ['t1Contrast.9']
 ['T1cInterquartileRange.2']
 ['t1DifferenceVariance.7']
 ['t1Variance.8']
 ['T1cMedian.3']
 ['t1LargeDependenceHighGrayLevelEmphasis.1']
 ['t2RootMeanSquared.1']
 ['T1cContrast.2']
 ['t2Variance.6']
 ['T1c90Percentile.2']
 ['t1Imc1.6']
 ['T1cRootMeanSquared.2']
 ['t2LargeDependenceEmphasis.7']
 ['t1SumAverage.1']
 ['t1GrayLevelVariance.25']
 ['t1GrayLevelNonUniformityNormalized.16']
 ['t2LongRunEmphasis.8']
 ['t1HighGrayLevelRunEmphasis.1']
 ['t1MeanAbsoluteDeviation.7']
 ['T1cMaximumProbability.1']
 ['T1cMeanAbsoluteDeviation.2']
 ['t1MeanAbsoluteDeviation.8']
 ['T1cRobustMeanAbsoluteDeviation.1']
 ['T1cVariance.2']
 ['t1Id']
 ['T1cMeanAbsoluteDeviation.1']
 ['t1DifferenceEntropy.8']
 ['T1cInterquartileRange.1']
 ['t1LongRunHighGrayLevelEmphasis.7']
 ['T1cKurtosis']]

Heatmap of the model in the training samples:


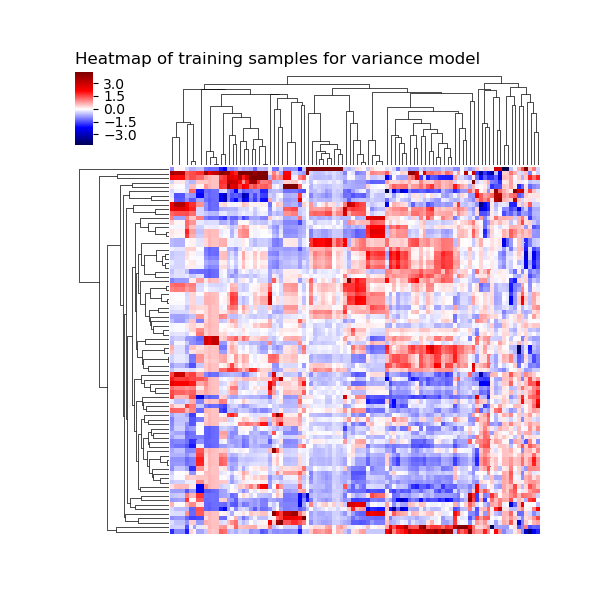


# 7. The method for selecting features: Correlation_xx

parameters setted: {'cutoff': 0.7}
num of remained features: 26
remained features:
[['T1cHighGrayLevelRunEmphasis.8']
 ['t1InverseVariance.8']
 ['t2HighGrayLevelZoneEmphasis']
 ['t2Uniformity.6']
 ['t2Variance']
 ['T1cSmallDependenceHighGrayLevelEmphasis.2']
 ['t1GrayLevelNonUniformityNormalized.15']
 ['t1Kurtosis.3']
 ['T1cKurtosis.2']
 ['t1Maximum.7']
 ['T1cGrayLevelVariance.9']
 ['t1Strength.7']
 ['t2Median.7']
 ['T1cIdmn.2']
 ['t1Autocorrelation.1']
 ['t2TotalEnergy.6']
 ['t2DifferenceAverage.1']
 ['t2LowGrayLevelZoneEmphasis.1']
 ['t1Variance.8']
 ['T1cMedian.3']
 ['T1cContrast.2']
 ['t2LongRunEmphasis.8']
 ['T1cMaximumProbability.1']
 ['t1Id']
 ['t1LongRunHighGrayLevelEmphasis.7']
 ['T1cKurtosis']]

Heatmap of the model in the training samples:


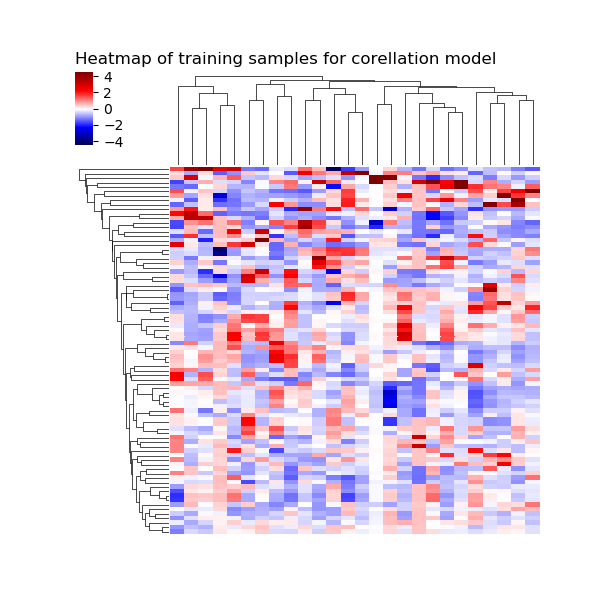


# 8. The method for selecting features: Univariate_Logistic

parameters setted: {'P value for threshold in': 0.05}
num of remained features: 11
remained features:
[['T1cMedian.3']
 ['t2DifferenceAverage.1']
 ['t1Kurtosis.3']
 ['t1GrayLevelNonUniformityNormalized.15']
 ['t2Median.7']
 ['t2LongRunEmphasis.8']
 ['T1cIdmn.2']
 ['T1cKurtosis.2']
 ['T1cKurtosis']
 ['t1Maximum.7']
 ['T1cMaximumProbability.1']]

Heatmap of the model in the training samples:


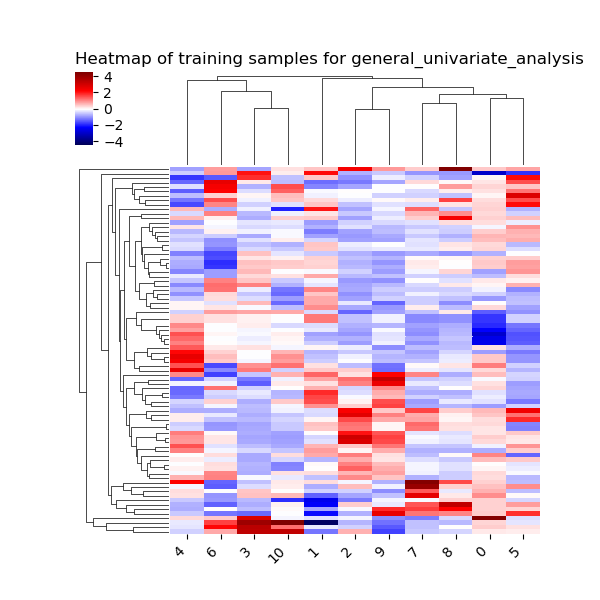


# 9. The method for selecting features: MultiVariate_Logistic

parameters setted: {'P value for threshold in': 0.05, 'P value for threshold out': 0.1}
**num of remained features: 5**
remained features:
[['t1GrayLevelNonUniformityNormalized.15']
 ['T1cIdmn.2']
 ['T1cKurtosis.2']
 ['t1Kurtosis.3']
 ['T1cMedian.3']]

Heatmap of the model in the training samples:


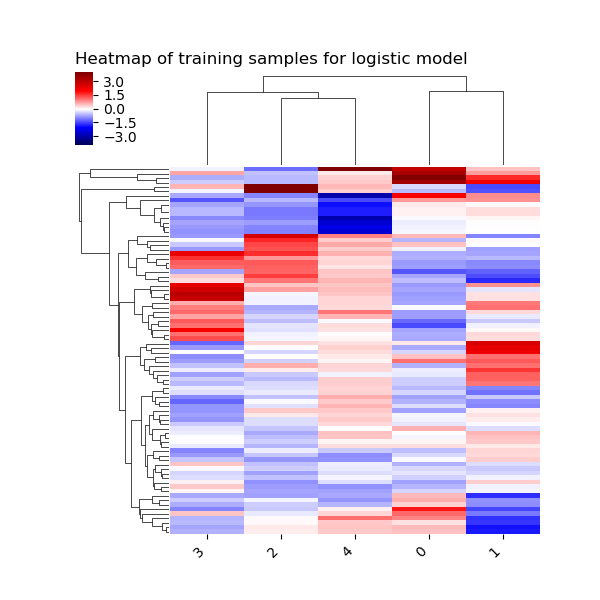

Supplement: Supplementary file 2 [file DataSheet_2.doc]
