# Supplementary material for: Multiparametric MRI Radiomics for the Early Prediction of Response to Chemoradiotherapy in Patients With Postoperative Residual Gliomas: An Initial Study
Source: Front Oncol. 2021 Nov 18;11:779202. doi: 10.3389/fonc.2021.779202 (PMC8636428; doi:10.3389/fonc.2021.779202)

**Statistical Report**

Author: DESKTOP-LLEB9HD

Data: 'train_T1-w.csv'

# Summary Report

# 1. Data: C:/Users/Dell-EFY/Desktop/data/train_T1-w.csv

# 2. Random seed: 123

# 3. Seperative rate: 1.0

Seperated report:

|  | Sum | Pos | Neg |
| --- | --- | --- | --- |
| data | 82 | 41 | 41 |
| train | 82 | 41 | 41 |
| test | 0 | 0 | 0 |

# 4. The method for standardizing the data: Standardization

# 5. The method for selecting features: General_Univariate_analysis

parameters setted: {'P value for threshold in': 0.05}
num of remained features: 99
remained features:
[['t1ClusterProminence.6']
 ['t1Idmn.4']
 ['t1Contrast.4']
 ['t1SumEntropy']
 ['t1ClusterTendency.2']
 ['t1Correlation']
 ['t1InverseVariance.7']
 ['t1DifferenceEntropy.6']
 ['t1Contrast.8']
 ['t1Idm.7']
 ['t1Idn.5']
 ['t1Complexity.8']
 ['t1ClusterProminence.7']
 ['t1Imc1.6']
 ['t1GrayLevelNonUniformityNormalized.15']
 ['t1Id.2']
 ['t1Complexity.2']
 ['t1ClusterTendency.6']
 ['t1Contrast']
 ['t1Range.8']
 ['t1Id.4']
 ['t1Idmn.5']
 ['t1DifferenceAverage.5']
 ['t1Median.6']
 ['t1Id.7']
 ['t1Idm.4']
 ['t1DifferenceAverage.2']
 ['t1Imc2.6']
 ['t1InverseVariance']
 ['t1InverseVariance.6']
 ['t1Maximum.7']
 ['t1LargeDependenceEmphasis.7']
 ['t1Id']
 ['t1Complexity.5']
 ['t1Contrast.10']
 ['t1ClusterProminence.2']
 ['t1ClusterTendency']
 ['t1ClusterProminence.4']
 ['t1Correlation.5']
 ['t1Strength.7']
 ['t1Idn.2']
 ['t1DependenceNonUniformityNormalized.7']
 ['t1ClusterShade.7']
 ['t1Contrast.5']
 ['t1ClusterProminence.5']
 ['t1DifferenceEntropy.3']
 ['t1DifferenceAverage.4']
 ['t1Idm.2']
 ['t1DifferenceAverage.6']
 ['t1Maximum.1']
 ['t1Skewness.8']
 ['t1GrayLevelVariance.25']
 ['t1DifferenceAverage']
 ['t1SumEntropy.2']
 ['t1InverseVariance.5']
 ['t1Id.5']
 ['t1JointEnergy.6']
 ['t1Id.6']
 ['t1GrayLevelVariance.22']
 ['t1ClusterTendency.1']
 ['t1JointEntropy.6']
 ['t1InverseVariance.2']
 ['t1Maximum.8']
 ['t1Autocorrelation.4']
 ['t1Correlation.2']
 ['t1Contrast.9']
 ['t1Skewness.7']
 ['t1Idn.4']
 ['t1Complexity.4']
 ['t1Idm']
 ['t1SumEntropy.6']
 ['t1GrayLevelVariance.23']
 ['t1Imc2.1']
 ['t1DifferenceAverage.7']
 ['t1ClusterProminence']
 ['t1Correlation.4']
 ['t1Idm.5']
 ['t1Contrast.11']
 ['t1ClusterProminence.8']
 ['t1Contrast.2']
 ['t1Idmn.2']
 ['t1LongRunHighGrayLevelEmphasis.7']
 ['t1MCC.6']
 ['t1InverseVariance.4']
 ['t1Correlation.6']
 ['t1ClusterTendency.5']
 ['t1Complexity.7']
 ['t1Idmn.7']
 ['t1GrayLevelNonUniformityNormalized.16']
 ['t1DifferenceVariance.6']
 ['t1DifferenceVariance.3']
 ['t1InverseVariance.1']
 ['t1SumEntropy.1']
 ['t1MaximumProbability.6']
 ['t1Median.2']
 ['t1Idm.6']
 ['t1ClusterTendency.4']
 ['t1Contrast.12']
 ['t1Range.7']]

Heatmap of the model in the training samples:


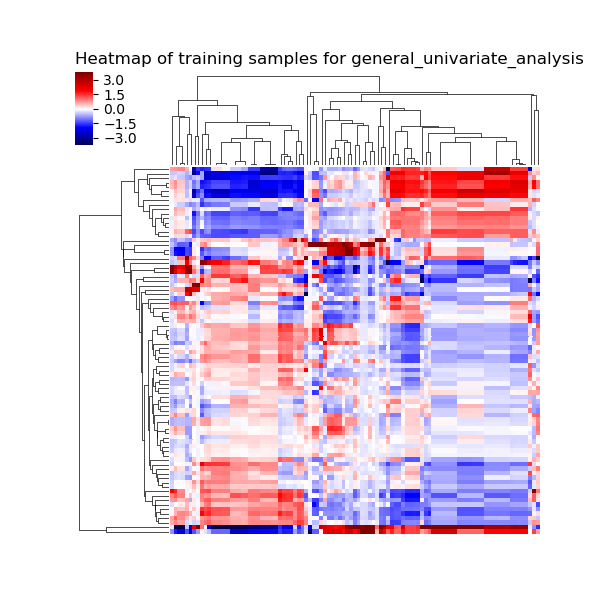


# 6. The method for selecting features: Variance

parameters setted: {'threshold': 1.0}
num of remained features: 43
remained features:
[['t1Idmn.4']
 ['t1Contrast.4']
 ['t1Correlation']
 ['t1InverseVariance.7']
 ['t1DifferenceEntropy.6']
 ['t1Complexity.8']
 ['t1Imc1.6']
 ['t1Id.2']
 ['t1ClusterTendency.6']
 ['t1Range.8']
 ['t1DifferenceAverage.2']
 ['t1InverseVariance.6']
 ['t1ClusterProminence.4']
 ['t1Correlation.5']
 ['t1Contrast.5']
 ['t1Idm.2']
 ['t1DifferenceAverage.6']
 ['t1Maximum.1']
 ['t1Skewness.8']
 ['t1GrayLevelVariance.25']
 ['t1DifferenceAverage']
 ['t1SumEntropy.2']
 ['t1Id.5']
 ['t1JointEnergy.6']
 ['t1Id.6']
 ['t1JointEntropy.6']
 ['t1InverseVariance.2']
 ['t1Correlation.2']
 ['t1Contrast.9']
 ['t1Idn.4']
 ['t1Imc2.1']
 ['t1Idm.5']
 ['t1Contrast.11']
 ['t1Contrast.2']
 ['t1MCC.6']
 ['t1Idmn.7']
 ['t1DifferenceVariance.3']
 ['t1InverseVariance.1']
 ['t1SumEntropy.1']
 ['t1MaximumProbability.6']
 ['t1Median.2']
 ['t1ClusterTendency.4']
 ['t1Range.7']]

Heatmap of the model in the training samples:


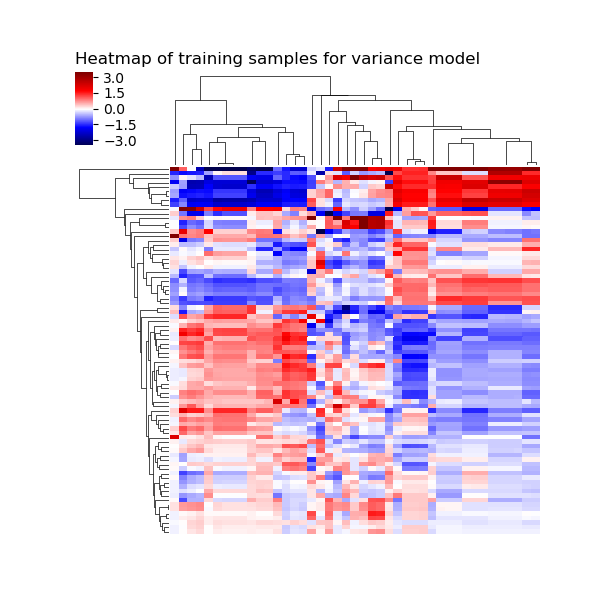


# 7. The method for selecting features: Correlation_xx

parameters setted: {'cutoff': 0.7}
num of remained features: 9
remained features:
[['t1Maximum.1']
 ['t1Skewness.8']
 ['t1DifferenceAverage']
 ['t1SumEntropy.2']
 ['t1Imc2.1']
 ['t1Idmn.7']
 ['t1DifferenceVariance.3']
 ['t1SumEntropy.1']
 ['t1Median.2']]

Heatmap of the model in the training samples:


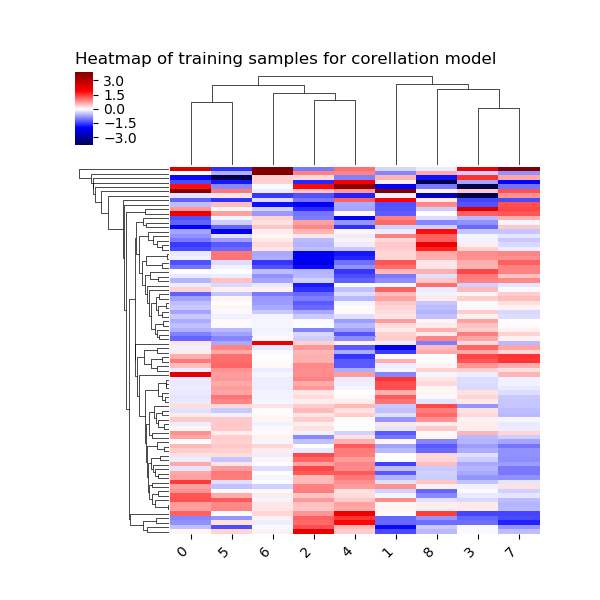


# 8. The method for selecting features: Univariate_Logistic

parameters setted: {'P value for threshold in': 0.05}
**num of remained features: 6**
remained features:
[['t1Idmn.7']
 ['t1Imc2.1']
 ['t1Skewness.8']
 ['t1DifferenceAverage']
 ['t1Median.2']
 ['t1SumEntropy.2']]

Heatmap of the model in the training samples:


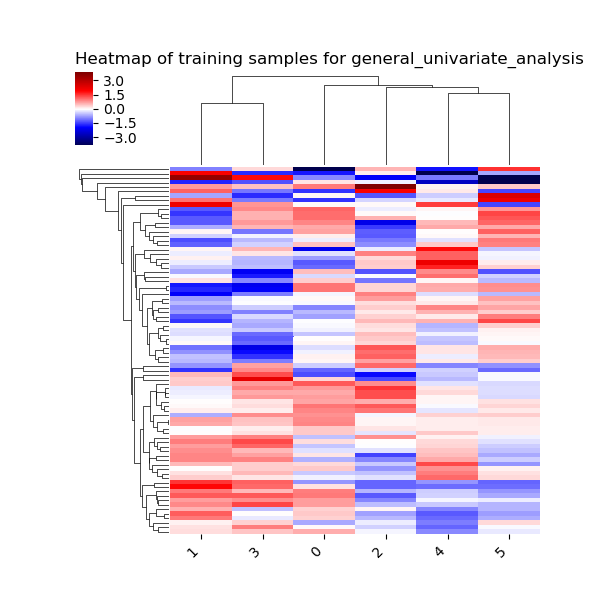

Supplement: Supplementary file 3 [file DataSheet_3.doc]
