# Supplementary material for: Multiparametric MRI Radiomics for the Early Prediction of Response to Chemoradiotherapy in Patients With Postoperative Residual Gliomas: An Initial Study
Source: Front Oncol. 2021 Nov 18;11:779202. doi: 10.3389/fonc.2021.779202 (PMC8636428; doi:10.3389/fonc.2021.779202)

**Statistical Report**

Author: DESKTOP-LLEB9HD

Data: 'train_CET1-w.csv'

# Summary Report

# 1. Data: C:/Users/Dell-EFY/Desktop/test(26case)/train_CET1-w.csv

# 2. Random seed: 16

# 3. Seperative rate: 1.0

Seperated report:

|  | Sum | Pos | Neg |
| --- | --- | --- | --- |
| data | 82 | 41 | 41 |
| train | 82 | 41 | 41 |
| test | 0 | 0 | 0 |

# 4. The method for standardizing the data: Standardization

# 5. The method for selecting features: General_Univariate_analysis

parameters setted: {'P value for threshold in': 0.05}
num of remained features: 57
remained features:
[['T1cKurtosis.4']
 ['T1cContrast.5']
 ['T1cUniformity.4']
 ['T1cSkewness.7']
 ['T1cRunVariance.6']
 ['T1c90Percentile.4']
 ['T1cEntropy.4']
 ['T1cKurtosis.6']
 ['T1cInterquartileRange.2']
 ['T1cSkewness']
 ['T1cVariance.5']
 ['T1c90Percentile.5']
 ['T1cRobustMeanAbsoluteDeviation.6']
 ['T1cSumSquares.3']
 ['T1cIdmn.2']
 ['T1cMCC.7']
 ['T1cInterquartileRange.5']
 ['T1c90Percentile.2']
 ['T1cInterquartileRange.1']
 ['T1cUniformity.3']
 ['T1cEntropy.3']
 ['T1cMeanAbsoluteDeviation.5']
 ['T1cKurtosis.5']
 ['T1cSkewness.8']
 ['T1cSmallAreaHighGrayLevelEmphasis.4']
 ['T1cRunVariance.1']
 ['T1cRobustMeanAbsoluteDeviation.5']
 ['T1cGrayLevelVariance.9']
 ['T1cRobustMeanAbsoluteDeviation.1']
 ['T1cKurtosis']
 ['T1cDifferenceVariance.6']
 ['T1cMedian.3']
 ['T1cImc1.6']
 ['T1cGrayLevelVariance.12']
 ['T1cKurtosis.2']
 ['T1cRootMeanSquared.2']
 ['T1cRobustMeanAbsoluteDeviation.3']
 ['T1c10Percentile.4']
 ['T1cRobustMeanAbsoluteDeviation.2']
 ['T1c10Percentile.2']
 ['T1cRobustMeanAbsoluteDeviation.4']
 ['T1cBusyness.6']
 ['T1cInterquartileRange.3']
 ['T1cMeanAbsoluteDeviation.4']
 ['T1cSmallDependenceHighGrayLevelEmphasis.2']
 ['T1cInterquartileRange.6']
 ['T1cVariance.2']
 ['T1cInterquartileRange']
 ['T1cDifferenceEntropy.6']
 ['T1cMeanAbsoluteDeviation.2']
 ['T1cIdn.2']
 ['T1cComplexity.2']
 ['T1cDependenceEntropy.3']
 ['T1c10Percentile.5']
 ['T1cHighGrayLevelZoneEmphasis.4']
 ['T1cRootMeanSquared.5']
 ['T1cInterquartileRange.4']]

Heatmap of the model in the training samples:


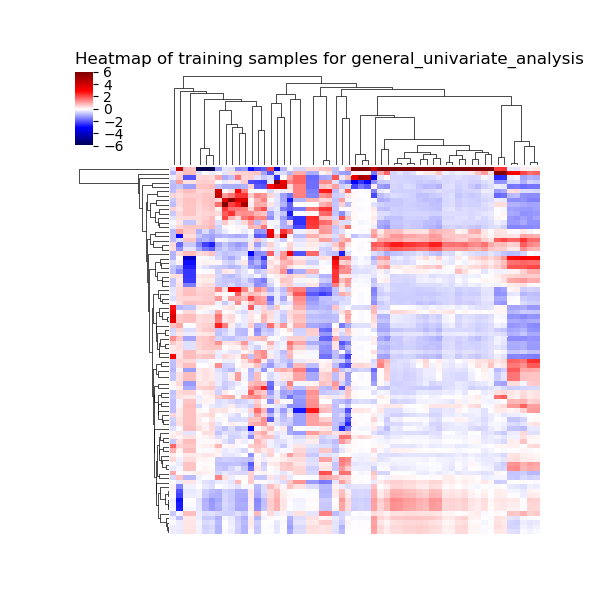


# 6. The method for selecting features: Variance

parameters setted: {'threshold': 1.0}
num of remained features: 24
remained features:
[['T1cKurtosis.4']
 ['T1c90Percentile.4']
 ['T1cEntropy.4']
 ['T1cKurtosis.6']
 ['T1cVariance.5']
 ['T1c90Percentile.5']
 ['T1cSumSquares.3']
 ['T1cIdmn.2']
 ['T1cInterquartileRange.5']
 ['T1cKurtosis.5']
 ['T1cSmallAreaHighGrayLevelEmphasis.4']
 ['T1cRunVariance.1']
 ['T1cRobustMeanAbsoluteDeviation.5']
 ['T1cDifferenceVariance.6']
 ['T1cImc1.6']
 ['T1cGrayLevelVariance.12']
 ['T1cKurtosis.2']
 ['T1c10Percentile.2']
 ['T1cRobustMeanAbsoluteDeviation.4']
 ['T1cInterquartileRange.3']
 ['T1cMeanAbsoluteDeviation.4']
 ['T1cVariance.2']
 ['T1cMeanAbsoluteDeviation.2']
 ['T1cHighGrayLevelZoneEmphasis.4']]

Heatmap of the model in the training samples:


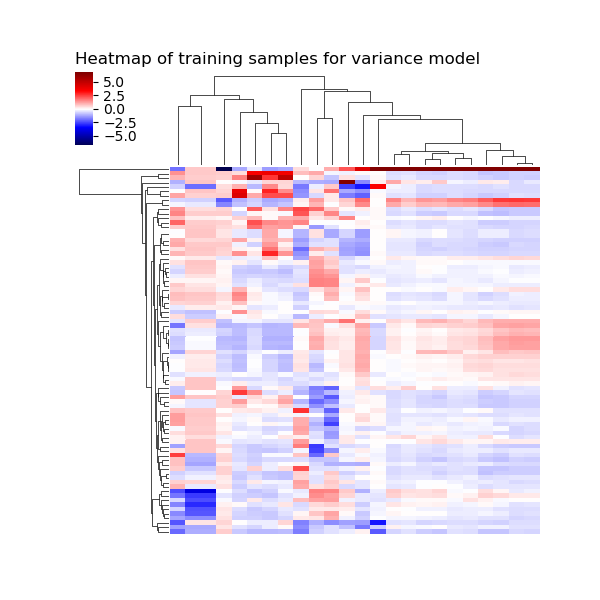


# 7. The method for selecting features: Correlation_xx

parameters setted: {'cutoff': 0.7}
num of remained features: 10
remained features:
[['T1cKurtosis.6']
 ['T1cSumSquares.3']
 ['T1cIdmn.2']
 ['T1cSmallAreaHighGrayLevelEmphasis.4']
 ['T1cRunVariance.1']
 ['T1cDifferenceVariance.6']
 ['T1cImc1.6']
 ['T1cGrayLevelVariance.12']
 ['T1cKurtosis.2']
 ['T1cHighGrayLevelZoneEmphasis.4']]

Heatmap of the model in the training samples:


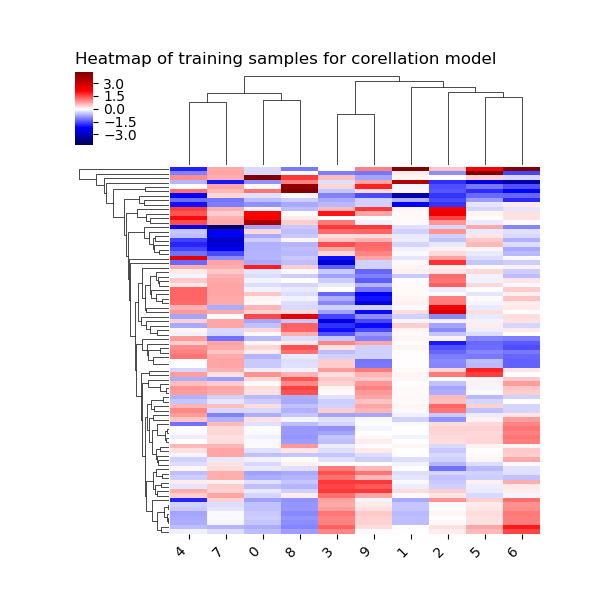


# 8. The method for selecting features: Univariate_Logistic

parameters setted: {'P value for threshold in': 0.05}
**num of remained features: 6**
remained features:
[['T1cRunVariance.1']
 ['T1cIdmn.2']
 ['T1cHighGrayLevelZoneEmphasis.4']
 ['T1cKurtosis.2']
 ['T1cSmallAreaHighGrayLevelEmphasis.4']
 ['T1cKurtosis.6']]

Heatmap of the model in the training samples:


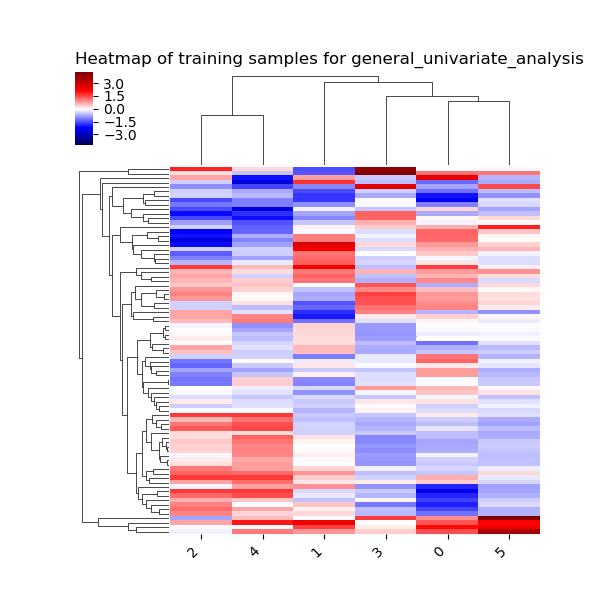

Supplement: Supplementary file 4 [file DataSheet_4.doc]
