# Supplementary material for: Multiparametric MRI Radiomics for the Early Prediction of Response to Chemoradiotherapy in Patients With Postoperative Residual Gliomas: An Initial Study
Source: Front Oncol. 2021 Nov 18;11:779202. doi: 10.3389/fonc.2021.779202 (PMC8636428; doi:10.3389/fonc.2021.779202)

**Statistical Report**

Author: DESKTOP-LLEB9HD

Data: 'train_T2-w.csv'

# Summary Report

# 1. Data: C:/Users/Dell-EFY/Desktop/data/train_T2-w.csv

# 2. Random seed: 123

# 3. Seperative rate: 1.0

Seperated report:

|  | Sum | Pos | Neg |
| --- | --- | --- | --- |
| data | 82 | 41 | 41 |
| train | 82 | 41 | 41 |
| test | 0 | 0 | 0 |

# 4. The method for standardizing the data: Standardization

# 5. The method for selecting features: General_Univariate_analysis

parameters setted: {'P value for threshold in': 0.05}
num of remained features: 162
remained features:
[['t2ShortRunLowGrayLevelEmphasis']
 ['t2JointEntropy.7']
 ['t210Percentile']
 ['t2Strength.7']
 ['t2HighGrayLevelZoneEmphasis.6']
 ['t2ShortRunHighGrayLevelEmphasis.6']
 ['t2Entropy.6']
 ['t2TotalEnergy.6']
 ['t2Range.7']
 ['t2Energy.5']
 ['t2GrayLevelVariance.18']
 ['t2Id.7']
 ['t290Percentile']
 ['t2JointEnergy']
 ['t2RootMeanSquared']
 ['t2Maximum.8']
 ['t210Percentile.2']
 ['t2Id.8']
 ['t2ClusterShade.8']
 ['t2RootMeanSquared.3']
 ['t2Maximum.2']
 ['t2Uniformity.1']
 ['t2ShortRunEmphasis.8']
 ['t2Variance']
 ['t2RootMeanSquared.1']
 ['t2GrayLevelVariance']
 ['t2DifferenceAverage.7']
 ['t2Variance.6']
 ['t2Uniformity']
 ['t2ClusterTendency.1']
 ['t2SumEntropy.6']
 ['t2DependenceEntropy.8']
 ['t2RootMeanSquared.2']
 ['t2LowGrayLevelEmphasis']
 ['t2Range.8']
 ['t2ClusterProminence.7']
 ['t2Variance.1']
 ['t2LowGrayLevelRunEmphasis']
 ['t2RunPercentage.8']
 ['t2SumSquares.6']
 ['t2RunLengthNonUniformityNormalized.8']
 ['t2HighGrayLevelEmphasis.6']
 ['t2DifferenceVariance']
 ['t2DifferenceVariance.6']
 ['t2MeanAbsoluteDeviation.1']
 ['t2ShortRunHighGrayLevelEmphasis.7']
 ['t2JointEntropy.8']
 ['t2Range.2']
 ['t290Percentile.4']
 ['t210Percentile.5']
 ['t2Contrast']
 ['t2Contrast.16']
 ['t2ClusterTendency.6']
 ['t2MeanAbsoluteDeviation.3']
 ['t2SumEntropy.1']
 ['t2DifferenceVariance.8']
 ['t2DifferenceAverage.1']
 ['t2LargeDependenceEmphasis.8']
 ['t2SmallDependenceHighGrayLevelEmphasis.8']
 ['t2Variance.3']
 ['t2DifferenceEntropy.1']
 ['t2InterquartileRange']
 ['t2DifferenceEntropy.8']
 ['t2HighGrayLevelRunEmphasis.7']
 ['t2SumSquares']
 ['t2ClusterTendency']
 ['t2HighGrayLevelZoneEmphasis.7']
 ['t2Contrast.1']
 ['t2GrayLevelNonUniformityNormalized.2']
 ['t2HighGrayLevelZoneEmphasis.8']
 ['t2GrayLevelVariance.4']
 ['t2JointEnergy.1']
 ['t2GrayLevelVariance.19']
 ['t2Contrast.14']
 ['t2DependenceNonUniformityNormalized.8']
 ['t2Complexity.1']
 ['t2RobustMeanAbsoluteDeviation.1']
 ['t2SmallDependenceHighGrayLevelEmphasis.7']
 ['t2Uniformity.6']
 ['t2InverseVariance.8']
 ['t2LowGrayLevelZoneEmphasis']
 ['t2DifferenceVariance.1']
 ['t2Variance.5']
 ['t2SumAverage']
 ['t2Entropy']
 ['t2GrayLevelVariance.1']
 ['t2SumSquares.1']
 ['t2ClusterProminence.6']
 ['t2RootMeanSquared.6']
 ['t2Idn.8']
 ['t290Percentile.2']
 ['t210Percentile.4']
 ['t2RootMeanSquared.4']
 ['t2Entropy.1']
 ['t2MeanAbsoluteDeviation.6']
 ['t2LargeDependenceEmphasis.1']
 ['t2LongRunHighGrayLevelEmphasis.8']
 ['t2JointEntropy']
 ['t2InverseVariance.7']
 ['t2RobustMeanAbsoluteDeviation']
 ['t290Percentile.1']
 ['t210Percentile.6']
 ['t2Id.1']
 ['t2DifferenceEntropy']
 ['t2LongRunEmphasis.7']
 ['t290Percentile.6']
 ['t2Strength.6']
 ['t2MeanAbsoluteDeviation.4']
 ['t2SumEntropy']
 ['t2RootMeanSquared.5']
 ['t2MeanAbsoluteDeviation']
 ['t2Variance.4']
 ['t2ShortRunHighGrayLevelEmphasis.8']
 ['t2InterquartileRange.6']
 ['t2Maximum.7']
 ['t2Complexity.3']
 ['t2RunVariance.8']
 ['t2RobustMeanAbsoluteDeviation.6']
 ['t2Minimum.4']
 ['t2MeanAbsoluteDeviation.2']
 ['t2MeanAbsoluteDeviation.5']
 ['t2TotalEnergy.5']
 ['t2LargeDependenceEmphasis.7']
 ['t2RunVariance.7']
 ['t2Idm.8']
 ['t210Percentile.1']
 ['t2GrayLevelVariance.3']
 ['t2RunPercentage.7']
 ['t2Energy.6']
 ['t2HighGrayLevelZoneEmphasis']
 ['t2RunLengthNonUniformityNormalized.7']
 ['t290Percentile.5']
 ['t2RunPercentage.1']
 ['t2Complexity.8']
 ['t2Idm.1']
 ['t2DifferenceAverage.8']
 ['t2Maximum.1']
 ['t2JointEntropy.1']
 ['t2LargeAreaLowGrayLevelEmphasis']
 ['t2GrayLevelNonUniformityNormalized']
 ['t2Complexity.7']
 ['t2Imc1.7']
 ['t2JointEntropy.6']
 ['t2JointAverage']
 ['t2Contrast.12']
 ['t2ClusterProminence.1']
 ['t2Variance.2']
 ['t2ClusterProminence.8']
 ['t2DifferenceVariance.7']
 ['t2DifferenceEntropy.7']
 ['t2InterquartileRange.1']
 ['t2Idm.7']
 ['t2HighGrayLevelRunEmphasis.6']
 ['t2ShortRunEmphasis.7']
 ['t2SmallDependenceEmphasis.7']
 ['t2GrayLevelNonUniformityNormalized.12']
 ['t2DifferenceEntropy.6']
 ['t2ClusterShade.7']
 ['t2Contrast.2']
 ['t2Idmn']
 ['t2LargeDependenceLowGrayLevelEmphasis']
 ['t2LongRunEmphasis.8']]

Heatmap of the model in the training samples:


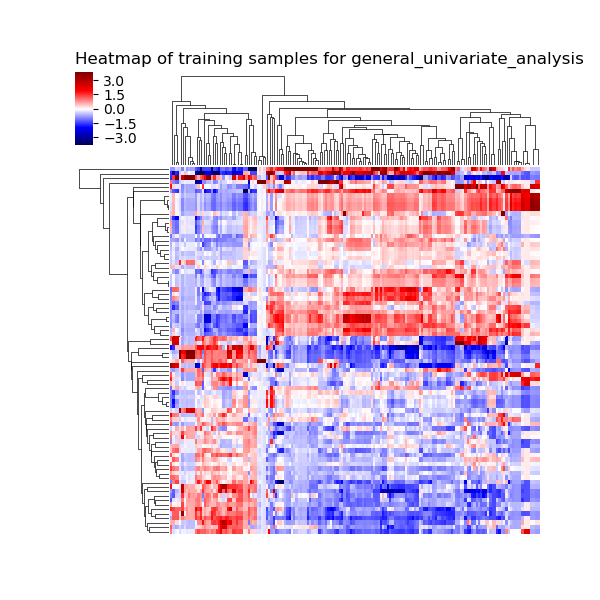


# 6. The method for selecting features: Correlation_xx

parameters setted: {'cutoff': 0.7}
num of remained features: 19
remained features:
[['t2Strength.7']
 ['t2Maximum.2']
 ['t2SumEntropy.6']
 ['t2DifferenceVariance']
 ['t2Contrast.1']
 ['t2Variance.5']
 ['t2Idn.8']
 ['t2LargeDependenceEmphasis.1']
 ['t2LongRunHighGrayLevelEmphasis.8']
 ['t2Strength.6']
 ['t2Complexity.3']
 ['t2Minimum.4']
 ['t2TotalEnergy.5']
 ['t2Maximum.1']
 ['t2LargeAreaLowGrayLevelEmphasis']
 ['t2Imc1.7']
 ['t2Contrast.12']
 ['t2HighGrayLevelRunEmphasis.6']
 ['t2ClusterShade.7']]

Heatmap of the model in the training samples:


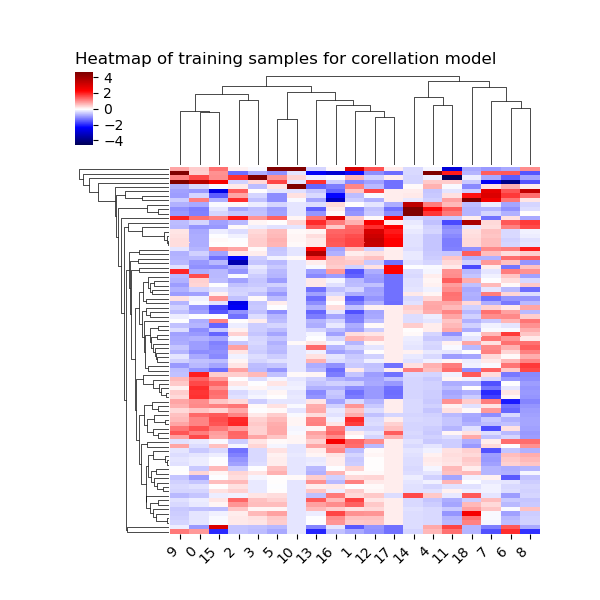


# 7. The method for selecting features: Univariate_Logistic

parameters setted: {'P value for threshold in': 0.05}
**num of remained features: 7**
remained features:
[['t2LargeDependenceEmphasis.1']
 ['t2LongRunHighGrayLevelEmphasis.8']
 ['t2SumEntropy.6']
 ['t2Idn.8']
 ['t2Contrast.12']
 ['t2Maximum.2']
 ['t2Imc1.7']]

Heatmap of the model in the training samples:


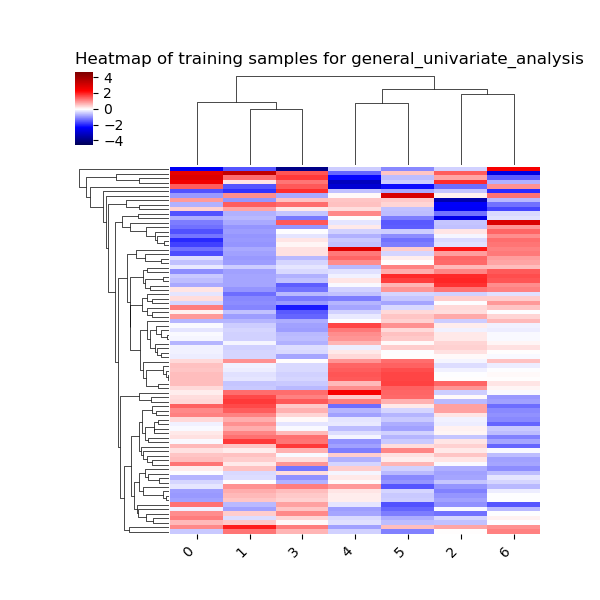

Supplement: Supplementary file 5 [file DataSheet_5.doc]
